# The Effect of High-Dose-Rate Pulsed Radiation on the Survival of Clinically Relevant Radioresistant Cells

**DOI:** 10.3390/life11121295

**Published:** 2021-11-25

**Authors:** Shingo Terashima, Hironori Yoshino, Yoshikazu Kuwahara, Hiro Sakuraba, Yoichiro Hosokawa

**Affiliations:** 1 Department of Radiation Science, Hirosaki University Graduate School of Health Sciences, Hirosaki 036-8564, Japan; hyoshino@hirosaki-u.ac.jp (H.Y.); hosokawa@hirosaki-u.ac.jp (Y.H.); 2 Department of Radiation Biology and Medicine, Faculty of Medicine, Tohoku Medical and Pharmaceutical University, Sendai 983-8536, Japan; y-kuwahara@tohoku-mpu.ac.jp; 3 Department of Applied Pharmacology, Graduate School of Medical and Dental Sciences, Kagoshima University, Kagoshima 890-8544, Japan; 4 Department of Radiology, Aomori City Hospital, Aomori 030-0821, Japan; haikyuu1231@gmail.com

**Keywords:** radiation therapy, clinically relevant radioresistant cells, γH2AX, pulsed radiation, ROS

## Abstract

We demonstrated that low dose pulsed radiation (0.25 Gy) at a high-dose-rate, even for very short intervals (10 s), decreases cell survival to a greater extent than single exposure to a similar total dose and dose rate. The objective of this study was to clarify whether high-dose-rate pulsed radiation is effective against SAS-R, a clinically relevant radioresistant cell line. Cell survival following high-dose-rate pulsed radiation was evaluated via a colony assay. Flow cytometry was utilized to evaluate γH2AX, a molecular marker of DNA double-strand breaks and delayed reactive oxygen species (ROS) associated with radiation-induced apoptosis. Increased cytotoxicity was observed in SAS-R and parent SAS cells in response to high dose rate pulsed radiation compared to single dose, as determined by colony assays. Residual γH2AX in both cells subjected to high-dose-rate pulsed radiation showed a tendency to increase, with a significant increase observed in SAS cells at 72 h. In addition, high-dose-rate pulsed radiation increased delayed ROS more than the single exposure did. These results indicate that high-dose-rate pulsed radiation was associated with residual γH2AX and delayed ROS, and high-dose-rate pulsed radiation may be used as an effective radiotherapy procedure against radioresistant cells.

## 1. Introduction

Elimination of radioresistant cancer cells poses a serious issue in radiotherapy, because radioresistant cells, as well as hypoxic cells that are resistant to radiation, survive after radiation therapy, leading to cancer recurrence or metastasis [1,2]. Although a combination of anticancer drugs and radiosensitizers may be used to resolve these issues, the drugs used are often ineffective in practice, and the systemic side effects of these drugs are problematic. Thus, a method that is effective against radioresistant cells without involving drugs may improve the success rate of radiation therapy and ensure its success.

The low-dose region contains a radiosensitive region termed the hyper-radiosensitivity (HRS) region, and the application of low doses in radiotherapy to improve the radiosensitivity of cancer cells has been studied. Furthermore, increased radioresistance (IRR) is induced in the higher dose region. Administering successive doses with short (1–2 h) intervals in between led to a radioresistance-like adaptive response and an HRS recovery requiring at least a 3 h interval between successive doses [3]. Therefore, a technique termed Low Doses Fractionated Radiation Therapy (LDFRT) [4,5], in which low doses (<0.6 Gy) are delivered at intervals of several h, has been used. However, some studies [6,7] have reported that LDFRT did not show the desired enhancing effect. On the other hand, the feasibility of using LDFRT as a potentiator of chemotherapy has been studied, and the results of clinical trials have indicated improved efficacy [8]. In addition, the technique of pulsed low-dose rate radiotherapy (PLDR), which involves irradiation with 0.2 Gy at 3 min intervals for a total of 2 Gy, resulting in an apparent dose rate of 0.0667 Gy/min (total 2 Gy irradiation in 30 min), has been used for the purpose of re-irradiation [9,10]. PLDR [11] was developed as an irradiation method that involves applying HRS and reverses dose rate effects. PLDR associated clinical studies [12,13,14] have already been performed and reported as effective. Due to difficulties in assessing the effects of low doses, the mechanism underlying HRS remains unclear. It is generally believed that the mechanism of HRS is associated with impairment of double-strand break (DSB) DNA repair and G2/M cell cycle arrest, which induce cells to undergo apoptosis due to unrepaired DSB [8,15]. The irradiation protocols associated with multiple low dose irradiations have not yet been fully elucidated. HRS is said to be more strongly expressed in radioresistant cells [8,16]. Thus, the effects of multiple low dose irradiations on radioresistant cancer cells as well as the mechanisms underlying these effects remain unclarified.

We determined that multiple exposures to low-dose radiation (0.25 Gy) at high-dose-rates (≥1.5 Gy/min), even at short intervals of 10 s, effectively decreased cell survival compared with a single exposure at a similar total dose and dose rate [17]. Dose rates are important for irradiation protocols involving high-dose-rate pulsed radiation (high-dose-rate PR). When dose rates were lowered, the cytotoxic effects of high-dose-rate PR decreased. High-dose-rate PR is characterized by short periods of additional time required for irradiation, simplicity, and enhanced cytotoxic effects even when used singly. Therefore, it can be expected to improve the radiosensitivity of radioresistant cells. In this study, we evaluated the effects of high-dose-rate PR using SAS-R cells that were established as radioresistant cells at the Institute of Development, Aging and Cancer, Tohoku University. SAS-R cells are clinically relevant radioresistant (CRR) cells that exhibit radioresistance and continue to proliferate stably even after daily irradiation with 2 Gy [18,19].

On the other hand, high-dose-rate PR protocols are not consistent with the general mechanism of HRS and thus the mechanisms involved are unclear. In a previous study [17], high-dose-rate PR amounting to a total dose of 8 Gy reduced cell survival by less than approximately 40% compared with a single exposure, but no significant changes were observed in apoptosis or cell cycle analysis. Relatively high doses of HRS, such as 0.8 Gy, have been reported [4]. Moreover, significantly enhanced cytotoxic effects were reported to be associated with high-dose-rate PR, not only at low doses below a unit dose of 0.5 Gy, but also at maximum doses of approximately a unit dose of 2 Gy [17]. Furthermore, it is unclear why the IRR effect or a radioresistance-like adaptive response does not manifest when the cell lethal effect is enhanced by low-dose fractionated irradiation at very short time intervals [17,20]. Reportedly DNA DSBs are hypersensitive to low doses due to the HRS effect [21,22]. Furthermore, post-irradiation secondary production of reactive oxygen species (ROS) facilitates radiation-induced apoptotic signaling, and delayed ROS production induces DNA DSBs [23,24,25].

The purpose of this study was to examine the efficacy of the proposed high-dose-rate PR on radioresistant cancer cells and to elucidate its mechanism. We evaluated the effect of high-dose-rate PR on radioresistant cells via continuous irradiation for 5 d to mimic clinical conditions. In this study, irradiation up to a high dose of 8 Gy was performed, due to the small additional time required between irradiation in high-dose-rate PR. Further we evaluated γH2AX, an indicator of DNA DSBs, and intracellular ROS to determine the results of intracellular ROS and DNA DSB in high-dose-rate PR.

## 2. Materials and Methods

### 2.1. Cell Culture

Human oral squamous cell carcinoma cell line (SAS) was obtained from the Cell Resource Center for Biomedical Research, Institute of Development, Aging and Cancer, Tohoku University. Clinically relevant radioresistant SAS-R [18,19] cells were established from the parental cell line SAS. Cells were cultured in DMEM/Ham’s F-12 media (Pure Chemical Industries, Osaka, Japan), supplemented with 10% fetal bovine serum (FBS), and maintained at 37 °C with 95% air and 5% CO_2_. TrypLE Express (1X) (Thermo Fisher, Waltham, MA, USA) was used to dissociate cells from culture dishes.

### 2.2. X-Irradiation and Irradiation Schedule

**Figure 1:**
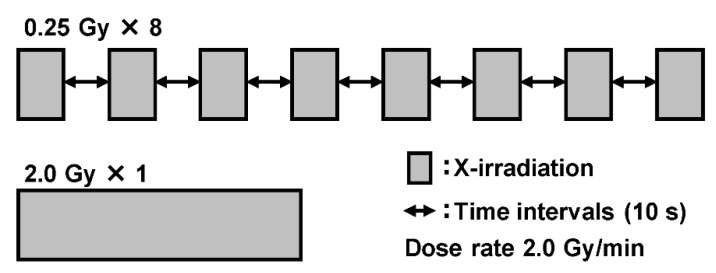
Schematic of the irradiation schedule. Cells were exposed to a unit dose of 0.25 Gy at 2.0 Gy/min with 10 s intervals.

X-irradiation was delivered using the MBR-1520R-3 X-ray machine (Hitachi Medico Technology, Tokyo, Japan) at 150 kVp through a 0.5 mm Al and 0.1 mm Cu filter. We performed fractionated exposures in unit doses of 0.25 Gy at a dose-rate of 2.0 Gy/min for 10 s intervals, as described previously [17]. Single exposure was delivered at the same dose rate. Error was less than 1 cGy even with a maximum estimate by one irradiation, since less than 1 cGy of the values indicated by the ionization chamber are omissions of fractions. If the maximum error associated with one irradiation is considered to be +1 cGy, with a uniform distribution of a B-type error, the propagation of error formula yields 0.25 Gy × 8 = 2.00 ± 0.008 Gy. A radiation schedule (2 Gy) scheme is shown as an example (Figure 1). For a total of 2 Gy, the irradiation time for a single exposure was 1 m 2 s, and the total irradiation time for high-dose-rate PR was 2 m 30 s. Due to the rising dose rate, an additional time of around 2 s was required for each irradiation, compared to the irradiation time calculated from the dose rate. The irradiation time of 0.25 Gy was considered to be 10 s. In order to mimic clinical conditions, an irradiation of 2 Gy per d for five consecutive days was performed to confirm that an enhanced cytotoxic effect could be maintained even with multiple low irradiation doses. Two days post plating, the cells were irradiated with 2 Gy per d for five consecutive days.

### 2.3. Cell Proliferation Assay

SAS and SAS-R cells were plated into two 35 mm culture dishes (IWAKI, Iwaki, Japan) at 2 × 10^4^ cells for each exposure. Two days post plating, the cells were irradiated with 2 Gy, 8 Gy, or 2 Gy per d consecutively for 5 d. The culture medium was changed 2 h prior to irradiation and 2 d after irradiation for 2 and 8 Gy, and 1 d and 3 d after the first irradiation for five consecutive days of irradiation (except for samples counted on the same day). Viable cells were counted using the trypan blue dye exclusion method at 0 h, 2, 4, and 6 d following the first irradiation.

### 2.4. Clonogenic Assay

Exponentially growing SAS (8.0 × 10^1^–1.0 × 10^4^) and SAS-R (8.0× 10^1^–6.0 × 10^3^) cells were plated onto four 60 mm culture dishes (IWAKI, Japan) for each exposure, depending on the dose of radiation. Next, the cells were irradiated 6 h later according to the above irradiation schedule before the cells began to replicate [26]. The experimental protocol that was used during five consecutive days of irradiation was similar to that of the cell viability assay, and the colony assay was performed 6 h after the final (fifth) irradiation. For 0 Gy, the cells were reseeded at 20,000/dish for 2 h prior to the second irradiation (1 d following the first irradiation). The colonies that were formed after incubation of SAS and SAS-R for 7–11 and 12–14 d, respectively, were stained with 4% Giemsa in PBS (-). Colonies containing more than 50 cells were considered as surviving cells. The fraction surviving each exposure was calculated as the ratio of plating efficiency for irradiated and unirradiated cells.

The surviving fraction data (0, 2, 4, 8 Gy) were fitted to the linear-quadratic (LQ) model, *lnS* = −α*D* − β*D*^2^, where *S* is the surviving fraction and *D* is the radiation dose using the Origin 8J program (Lightstone Corp., Tokyo, Japan). The least chi-square (χ^2^) fitting technique was used to analyze the data for nonlinear curve fitting. We did not allow α to be negative as a restriction [27]. The parameters, α, β, and *D*_10_ were calculated for each curve and the dose-modification factor at 10% survival (*DMF*_10_) was calculated as (*D*_10_ of single exposure)/(*D*_10_ of fractionated exposures).

A total of 100 radioresistant SAS-R cells in a 60 mm diameter dish are sufficient for 2 Gy irradiation. Due to the microdosimetric and statistical aspects associated with the small number of seedings, cell viability could not be sufficiently evaluated. We prepared 500,000 cell suspensions in SAS-R in a no-processing Φ35 mm dish (for Floating Cell); (IWAKI, Japan) to which cells do not adhere and re-seeded them under conditions of 200 cells/Φ60 mm dish after X-ray irradiation. Relative survival rates (normalized to single exposure) were calculated by dividing the survival rate of each sample by the arithmetic mean of the survival rate following single exposure and averaging these values. Thereafter, the colony assay was performed via the same protocol as above.

### 2.5. γH2AX Analysis by Flow Cytometry

In brief, cells were plated at 2 × 10^5^ cells onto 35 mm culture dishes. Twenty-four hours after plating, cells were exposed to 2 or 8 Gy. After a predetermined post-irradiation time point, collected cells were fixed in 70% ethanol and stored at −30 °C. Samples were fixed with ethanol and treated with anti-phospho-Histone H2A.X (Ser139) (Millipore, (Burlington, MA, USA)) at 1:500 in PBS (containing 0.1% FBS and 0.25% Triton-X 100) for 30 min at 37 °C. The samples were the incubated with Alexa Fluor 488-labeled goat anti-mouse IgG antibodies (Invitrogen, (Waltham, MA, USA)) at 1:500 in PBS (containing 0.1% FBS and 0.25% Triton-X 100) for 1 h at 37 °C. Samples were further incubated for 30 min at room temperature with 20 μg/mL propidium iodide (PI) and 100 μg/mL RnaseA in PBS (containing 0.1% FBS and 0.25% Triton-X 100). Samples were analyzed using a flow cytometer (Cytomics FC500; Beckman Coulter, Brea, CA, USA). Sample data were analyzed via Flowing Software (https://bioscience.fi/services/cell-imaging/flowing-software/ (accessed on 22 April 2021)).

### 2.6. Intracellular ROS Analysis by Flow Cytometry

Briefly, cells were plated at 2 × 10^5^ onto 35 mm culture dishes. Twenty-four hours after plating, cells were exposed to 8 Gy. To measure intracellular ROS production, three ROS detection reagents were used as follows: hydroxyphenyl fluorescein (HPF); Dihydrorhodamine 123 (DHR123); and dihydroethidium (DHE) (Molecular Probes, Invitrogen, Waltham, MA, USA). HPF [28] was designed to detect intracellular highly reactive oxygen species (hROS) such as the hydroxyl radical (^•^OH) and peroxynitrite (ONOO^−^) DHR123 is used to measure intracellular hydrogen peroxide (H_2_O_2_). DHE is an intracellular ROS indicator of superoxide (O2^•−^). Following 1, 2, and 3 d post irradiation, collected cells were incubated at 37 °C with 5 μM HPF, DHR123, or DHE in Hanks’ Balanced Salt Solutions (HBSS; with Ca^2+^ and Mg^2+^) for 30, 20, and 20 min, respectively. Samples were analyzed using a flow cytometer and Flowing Software.

### 2.7. Statistical Analysis

Statistical comparisons were performed using the Tukey–Kramer test for multiple comparisons and Welch’s *t*-test for two comparisons. Results are presented as arithmetic means ± standard deviation of data from at least three independent experiments. Statistical significance was set at *p* < 0.05. Statistical analyses were performed using Excel 2013 (Microsoft, (Redmond, Wash. USA)) with Statcel 3 add-in software (OMS Inc., Hamamatsu, Shizuoka, Japan).

## 3. Results

### 3.1. Cell Proliferation Assay

**Figure 2:**
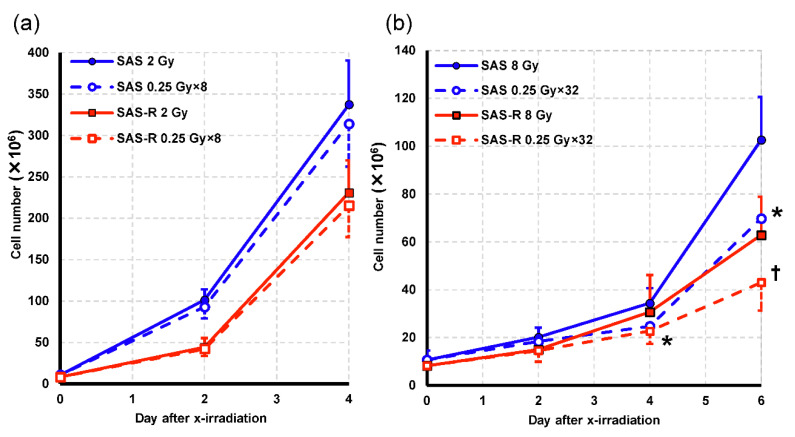
Changes in the viable cell number following X-irradiation at 2 Gy (**a**) and 8 Gy (**b**). Data represent the mean ± standard deviation of the results of three independent experiments, each with duplicate samples. * = *p* < 0.05 vs. SAS single exposure; † = *p* < 0.05 vs. SAS-R single exposure.

In the initial experiment, we evaluated the cell proliferation of SAS and SAS-R cells using fractionated exposures. Following 2 or 8 Gy irradiation, we counted viable SAS and SAS-R cells using the trypan blue dye exclusion method. The change in cellular proliferation is shown (Figure 2). For 2 Gy irradiation, the number of cells in SAS and SAS-R cells following irradiation via high-dose-rate PR was slightly lower than that via single exposure up to 4 d, and no significant difference was observed between the two procedures. Four days following 8 Gy irradiation, the cell number in SAS cells exposed to high-dose-rate PR was significantly decreased compared with SAS cells exposed to single exposure (*p* = 0.011). At 6 d post-irradiation, the cell number in both SAS and SAS-R subjected to high-dose-rate PR was significantly decreased compared to that subjected to single exposure (*p* = 0.013 and *p* = 0.04, respectively).

**Figure 3:**
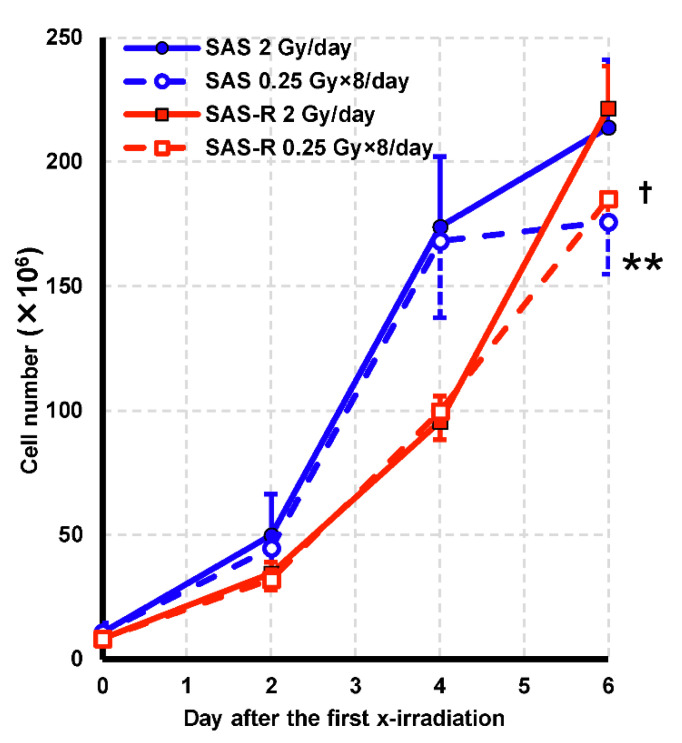
The change in viable cell number due to irradiation at 2 Gy/day for 5 d is shown. Data are represented by the mean ± standard deviation of the results of three independent experiments, each with duplicate samples. ** = *p* < 0.01 vs. SAS single exposure; † = *p* < 0.05 vs. SAS-R single exposure.

Next, we evaluated the effect of high-dose-rate PR at a daily irradiation dose of 2 Gy for five consecutive days, which mimics clinical radiotherapy conditions. The viable cell number following five consecutive days of daily irradiation at 2 Gy is shown (Figure 3). At the end of the fifth irradiation (at day 4 following the first irradiation), no significant difference in cell number was observed in both cells. On day 6 (day 2 after the end of 5 d of irradiation), the number of cells in SAS and SAS-R exposed to high-dose-rate PR was significantly decreased compared with that for single exposure (*p* = 0.008 and *p* = 0.03, respectively).

### 3.2. Clonogenic Assay

**Figure 4:**
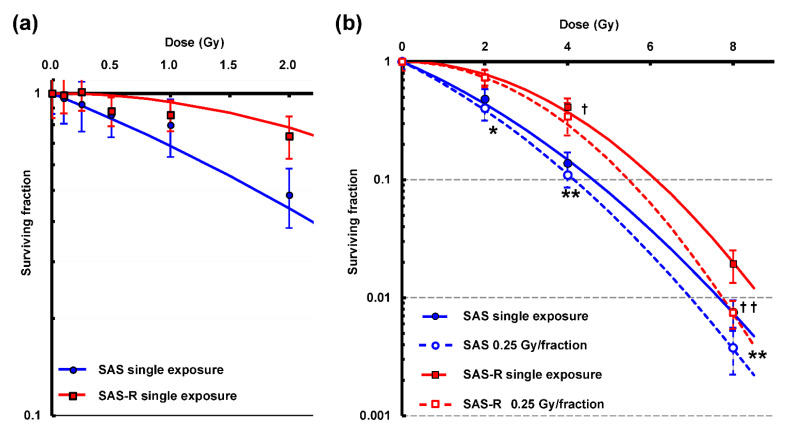
Cell survival curves of SAS and SAS-R cells subjected to a single exposure or high-dose-rate PR. Results of survival fraction at 0, 0.1, 0.25, 0.5, 1, and 2 Gy (**a**) and at 0, 2, 4, and 8 Gy (**b**) are shown. Curves were fitted to the linear–quadratic model using surviving fraction data (0, 2, 4, and 8 Gy) in (**a**,**b**). Data are represented by the mean ± standard deviation of three to five independent experiments, each with four samples. * = *p* < 0.05 vs. SAS single exposure; ** = *p* < 0.01 vs. SAS single exposure; † = *p* < 0.05 vs. SAS-R single exposure; †† = *p* < 0.01 vs. SAS-R single exposure.

The results of the colony assay of SAS and SAS-R are shown (Figure 4). The cell survival rate for single exposure below 2 Gy is presented (Figure 4a). Although the results are somewhat suggestive of the presence of HRS and IRR in both cells, these survival fraction values almost coincided with LQ model curves when standard deviation was taken into consideration (excluding SAS-R 0.5 Gy). Even at 0.25 Gy, which is the unit dose used in high-dose-rate PR, survival in both cell types was not significantly decreased. Survival curves fitted to the LQ model showed that SAS-R was more resistant to radiation than SAS, and that cell survival was significantly higher in SAS-R than in SAS at doses of 2 Gy and higher (*p* < 0.001). The cell survival rate for high-dose-rate PR was significantly lower than that for single exposure at 2, 4, and 8 Gy, except for SAS-R at 2 Gy (Figure 4b). Results of the colony assay for 2 Gy irradiation of 500,000 cell suspensions on a no processing Φ35 mm dish, showed that relative survival in SAS-R (normalized to 2 Gy single exposure) subjected to high-dose-rate PR was significantly lower than that corresponding to single exposure (*p* < 0.001). Relative survivals corresponding to single exposure and high-dose-rate PR were 1.0 ± 0.13 and 0.78 ± 0.16, respectively.

Parameters of α and β, as well as α/β, *D*_10_ and *DMF*_10_, calculated from these parameters, are shown (Table 1). Considering the parameters of SAS and SAS-R, a characteristic feature was that the α value of SAS-R was close to 0, and consequently the α/β value was also close to 0 (α, α/β = 0 in high-dose-rate PR). Comparing single exposure and high-dose-rate PR, we observed a slight increase in the β values of both cell lines. In high-dose-rate PR, *DMF*_10_ was approximately 1.1 for both SAS and SAS-R cells.

**Figure 5:**
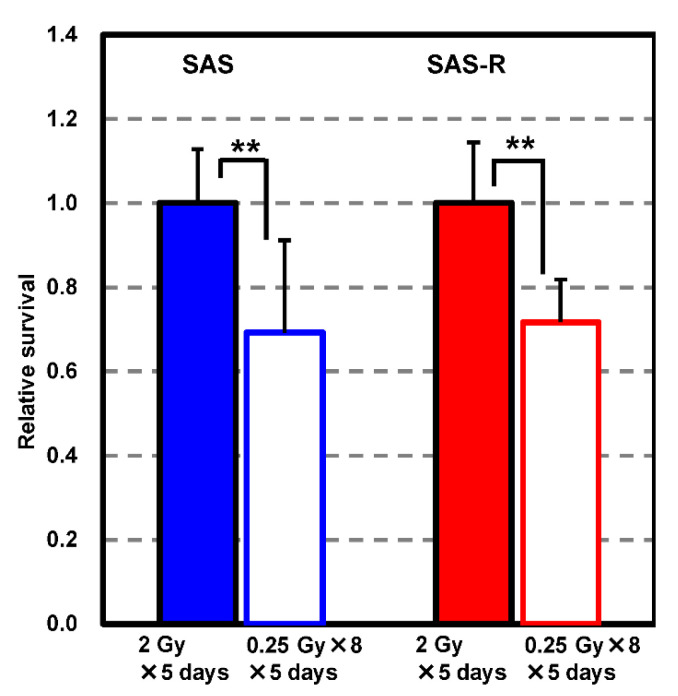
Relative survival was evaluated via clonogenic assaying of SAS and SAS-R cells after 2 Gy/day (single exposure or high-dose-rate PR) for five consecutive days. Data represent the mean ± standard deviation of the results of four to five independent experiments, each with four samples. ** = *p* < 0.01.

The relative survival of SAS and SAS-R cells was significantly lower for high-dose-rate PR than that for single exposure at 2 Gy/day for five consecutive days (*p* < 0.001); (Figure 5). The survival rates of SAS and SAS-R cells after five consecutive days of irradiation were 0.024 ± 0.0097 and 0.19 ± 0.12, respectively. Thus, SAS-R showed strong radioresistance in five consecutive days irradiation.

### 3.3. γH2AX Analysis by Flow Cytometry

**Figure 6:**
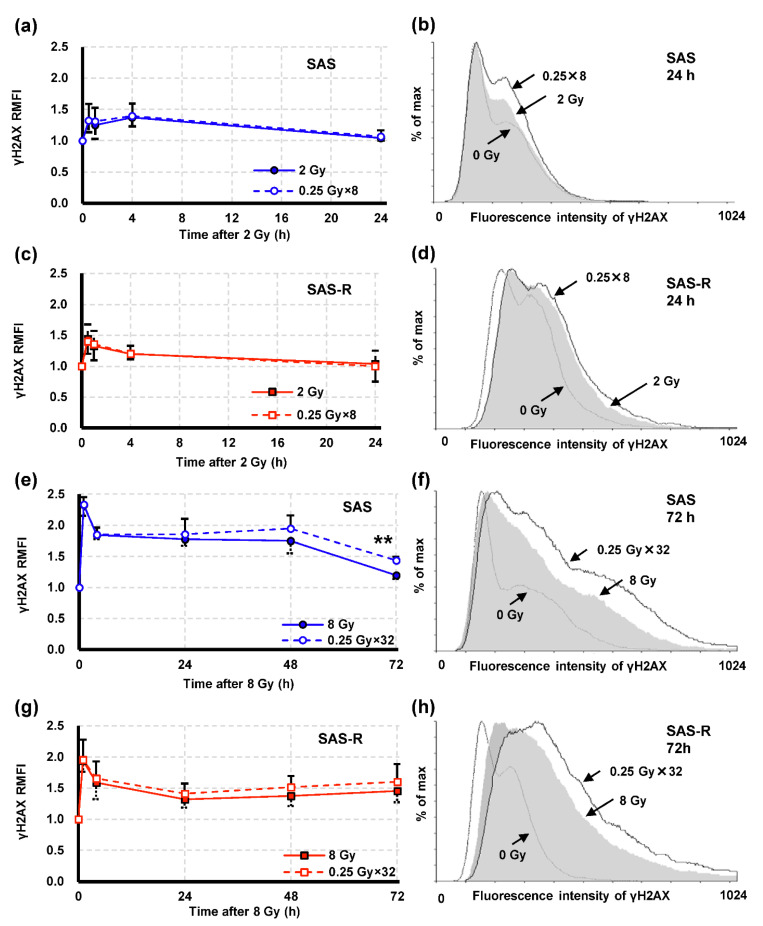
γH2AX analysis via flow cytometry. Kinetics of γH2AX relative median fluorescence intensity (RMFI) (normalized to 0 h control) of SAS ((**a**); 2 Gy, (**e**); 8 Gy) and SAS-R ((**c**); 2 Gy, (**g**); 8 Gy); (normalized to control (0 h)). A representative flow cytometric histogram showing γH2AX fluorescence intensity of SAS ((**b**); 2 Gy, (**f**); 8 Gy) and SAS-R ((**d**); 2 Gy, (**h**); 8 Gy) at 24 or 72 h. Data are represented by the mean ± standard deviation of three to six independent experiments. ** = *p* < 0.01 vs. SAS single exposure.

γH2AX, a biomarker of DNA DSB, was analyzed using flow cytometry (Figure 6). For 2 Gy irradiation, relative median fluorescence intensity (RMFI) of γH2AX (normalized to 0 Gy at 0 h) was analyzed at 0.5, 1, 4, and 24 h after irradiation, but there was almost no difference between single exposure and high-dose-rate PR. We also examined the cell cycle and γH2AX levels in a cell cycle-dependent manner in both cell types, which revealed only a slight difference in γH2AX levels between single exposure and high-dose-rate PR (data not shown). In both cells, similar time-dependent induction of γH2AX was found for single exposure as well as high-dose-rate PR at 8 Gy. However, γH2AX RMFI in SAS cells subjected to high-dose-rate PR irradiation began to gradually increase from 24 h onwards and was significantly increased at 72 h (*p* = 0.007), compared with that for single exposure. Although, no significant increase in γH2AX RMFI was observed in high-dose-rate PR compared with single exposure in SAS-R cells, γH2AX RMFI corresponding to high-dose-rate PR continued to be higher than that corresponding to single exposure from 4 h onwards.

### 3.4. Intracellular ROS Analysis by Flow Cytometry

**Figure 7:**
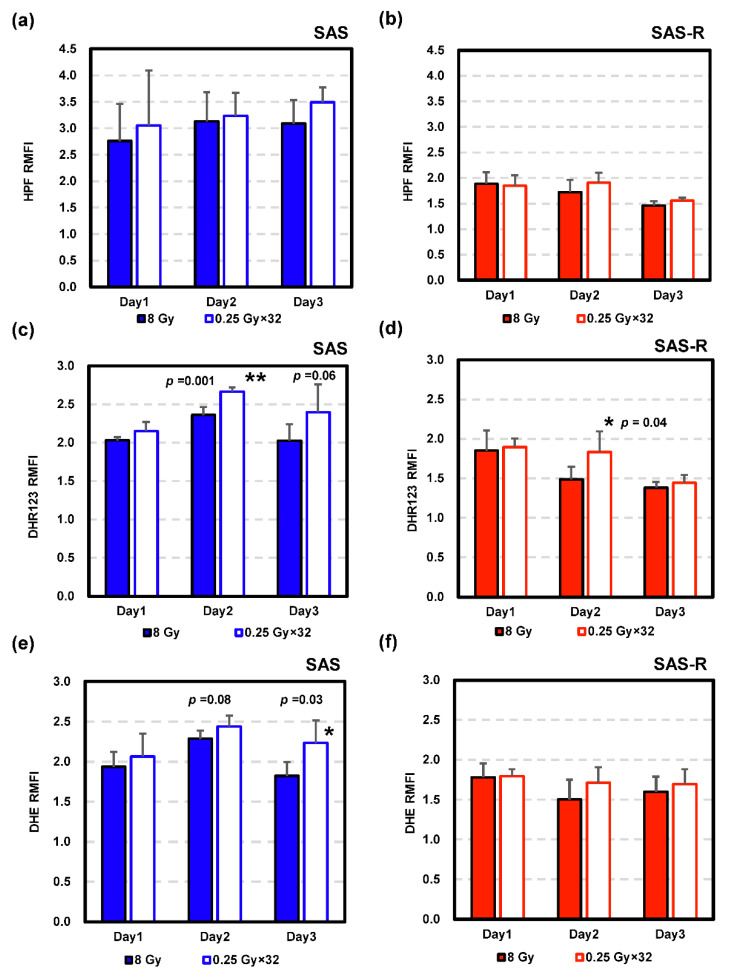
Evaluation of intracellular ROS levels analyzed via flow cytometry following X-irradiation. Cells were labeled with HPF (**a**,**b**), DHR123 (**c**,**d**), or DHE (**e**,**f**). Relative median fluorescence intensity (RMFI) was normalized to control (0 Gy) at the corresponding time points. Data are represented by the mean ± standard deviation of three to six independent experiments. * = *p* < 0.05 vs. single exposure; ** = *p* < 0.01 vs. single exposure.

RMFI of intracellular ROS levels obtained via flow cytometry using three probes are shown (Figure 7). In HPF, mainly OH and ONOO^−^ were measured. Whereas only a slight increase, with no significant change, in HPF RMFI was observed over time in SAS cells, a gradual decrease was observed in SAS-R. For both cells, no statistically significant difference was observed between single exposure and high-dose-rate PR. DHR123 is a fluorescent probe used to measure intracellular H_2_O_2_. A significant increase in DHR123 RMFI was observed in high-dose-rate PR compared with single exposure at 2 d after irradiation in SAS (*p* = 0.001). Three days after irradiation, DHR123 RMFI in SAS was also higher in high-dose-rate PR than in single exposure, although the difference was not statistically significant (*p* = 0.06). In SAS-R cells, RMFI in DHR123 tended to decrease with time, and 2 d after irradiation, RMFI in DHR123 in high-dose-rate PR was significantly higher than that in single exposure (*p* = 0.04). DHE was used to detect intracellular superoxide. DHE RMFI in SAS cells showed a similar trend to that of DHR123. In a comparison between DHE RMFI corresponding to a single exposure and high-dose-rate PR, the ROS level of high-dose-rate PR was slightly higher 2 d after irradiation (*p* = 0.08), and a significant increase was observed 3 d after irradiation (*p* = 0.03). On the other hand, no statistically significant difference was observed between single exposure and high-dose-rate PR in DHE RMFI in SAS-R cells.

## 4. Discussion

Recurrence of radioresistant cancer cells constitutes a serious issue in radiotherapy, and prevention of recurrence poses a challenge. In this study, we investigated whether our proposed method of high-dose-rate PR exerts enhanced cytotoxic effects on radioresistant cancer cells. We conducted a mechanistic analysis to evaluate γH2AX, a biomarker of DNA DSB, and intracellular ROS.

High-dose-rate PR exerted a significantly increased cytotoxic effect on radioresistant SAS-R and its parent SAS cells, in a dose dependent manner (Figure 4b). Compared with a single exposure, high-dose-rate PR exerted a significantly enhanced cytotoxic effect on SAS at 2 Gy but not on SAS-R. However, an additional colony assay using a no-processing dish indicated that high-dose-rate PR exerted a significantly increased cytotoxic effect compared with that by single exposure (*p* < 0.001). Our results suggested that the sensitivity of the assay at 2 Gy was reduced because SAS-R is radioresistant and the number of seeded cells in the conventional colony assay was low. In this experiment, we used 0.25 Gy as the unit dose for high-dose-rate PR. In both cell lines, cell survival at 0.25 Gy was almost similar on the survival curve of the LQ model, and no significant decrease in the survival rate was observed. It has been reported that HRS may be detected via a conventional colony assay if the experiment is performed with a high degree of accuracy [29]. HRS is generally detected via fluorescence-activated cell sorting and microscopy [30]. Todorovic et al. [10] reported that radioresistant cells were more sensitive to PLDR at a unit dose of 0.3 Gy, wherein radioresistant cells showed more HRS than its parental cells. However, they noted that the enhanced cytotoxic effect of PLDR disappeared at lower unit doses (0.2 Gy), and that HRS could not be observed in radioresistant cells. The *DMF*_10_ of high-dose-rate PR was 1.1 for both radioresistant cells and the parental cells in this experiment, and similar values were observed in our previous study with 1.14 and 1.18 for V79 and A549, respectively [17]. Although our results did not indicate that radioresistant cells were affected more by high-dose-rate PR, the high-dose-rate PR protocol was optimized and therefore effective against radioresistant cells as well as parental cells. In contrast, when comparing the change in *D*_10_ between the high-dose-rate PR and single exposure, the dose required for *D*_10_ was around 0.2 Gy lower in SAS-R compared to in SAS by using the high-dose-rate PR, indicating the greater enhancement of the cytotoxic effect in SAS-R.

The colony assay for radioresistant SAS-R cells did not indicate a significant difference between cytotoxic effects of high-dose-rate PR and a single irradiation of 2 Gy (Figure 4b). However, a significant increase in the cytotoxic effect was observed after five consecutive days of irradiation (Figure 3). Evaluation via the cell proliferation assay showed that high-dose-rate PR decreased the cell viability of SAS-R, whereas it proliferated stably when exposed to 2 Gy daily [19]. In Figure 2, the cell number and growth rate were higher in SAS than in SAS-R, because the doubling time of the logarithmic growth phase of SAS and SAS-R in this study was about 16 and 18 h, respectively. In addition, cells were seeded two days before irradiation, and the number of cells on the irradiation day was higher in SAS than in SAS-R. MTT assay involving five consecutive days of irradiation has also shown that PLDR not only decreased cell proliferation compared to single exposures, but also slowed cell proliferation [9]. Clinically, high-dose-rate PR at 2 Gy/day treatment was effective against radioresistant cells, whereas the 5-consecutive day irradiation experiments were undertaken to mimic clinical radiation therapy. Furthermore, final cell survival estimated via the colony assay showed an enhanced cytotoxic effect rate which was similar to that of the parental cells, and thus is expected to be utilized for clinical application (Figure 5).

High-dose-rate PR is a method that involves the use of multiple low dose irradiations at high dose rates. With a high dose rate, an enhanced cytotoxic effect can be obtained even with short intervals of 10 s, as evidenced in this study. While LDFRT and PLDR require multiple irradiations per day or a treatment time of about 30 min for irradiation alone, the advantage of high-dose-rate PR is that it requires irradiation only once-daily, and because the time intervals are short, the increase in treatment time per case is small. Thus, the load on staff and patients is lessened. In addition, high-dose-rate PR does not require any special equipment, and an enhanced cytotoxic effect can be achieved without using any drugs. Furthermore, since the additional time required by the irradiation protocol is short, it can be applied to large doses such as stereotactic radiotherapy.

The results of flow cytometry analysis revealed that the residual γH2AX levels were higher (indicating that DNA DSBs were not repaired) at high-dose-rate PR of 8 Gy than that at single exposure of 8 Gy. It has been reported that residual γH2AX after X-irradiation is an indicator of radiosensitivity, and that a correlation exists between residual γH2AX and cell survival [31,32]. No difference was observed in residual γH2AX at 2 Gy, because the sensitivity of flow cytometry was insufficient to detect γH2AX at lower dose (−0.3 Gy) [33]. At 8 Gy, there was almost no difference between high-dose-rate PR and single exposure at 1 and 4 h, but an increase in residual γH2AX levels was observed after 24 h. It is reported that slow progress in the repair process of DNA DSB indicates that DSB remains permanently unrepaired due to cellular senescence, apoptosis, or being unrepaired at specific genomic sequences, such as telomeres [34]. Wykes et al. [35] reported that failure to recognize DNA DSBs is not the result of HRS, and that a number of observations were associated with the DNA DSB repair pathway in response to HRS/IRR [8]. On the other hand, it has been proposed that the HRS effect is due to an increase in unrecognized DSBs at low doses because there is no translocation of ATM monomers from the cytoplasm to the nucleus and, as active ATM monomers do not phosphorylate H2AX, phosphorylation of H2AX does not occur [36,37]. Qvarnström et al. [22] reported that when detecting 53BP1 foci at 72 h in basal keratinocytes from biopsies, persistent 53BP1 foci above the baseline levels were still observed at 0.1 Gy but returned to baseline levels at higher doses, suggesting that HRS impairs efficient repair of DSBs. At 8 Gy irradiation, high-dose-rate PR increases residual DSBs at 72 h in SAS cells compared to single exposure, resulting in enhanced cytotoxic effects. It is suggested that multiple low doses of irradiation may inhibit sufficient DSB repair. Although a statistically significant increase in residual γH2AX was not observed for high-dose-rate PR in SAS-R cells, a similar increase in γH2AX was observed, suggesting a similar mechanism.

In regard to dose fractionation, another possible mechanism underlying high-dose-rate PR function is the Low Repeated Dose (LORD) effect. LORD involves the synergistic effect of two radiation doses, separated by a time interval, on the dynamics of chromatin recondensation linked to DSB repair [38]. The increase observed in residual γH2AX due to multiple irradiations, each delivered after a time interval according to the LORD effect, was consistent with the results of high-dose-rate PR. However, enhancement of the cytotoxic effect due to multiple irradiations, does not simply depend on the number of dose fractions, and the enhanced cytotoxic effect reportedly decreases when a larger fraction dose is first irradiated, even when the total dose and total number of fractions are the same (e.g., 1.0 + 0.5 + 0.5 Gy) [20,39]. Therefore, the mechanism of high-dose-rate PR is thought to be associated with both the effects of low doses, such as HRS, and the LORD effect.

In high-dose-rate PR, delayed intracellular ROS showed a greater increase than that in single exposure. Studies on HRS or low-dose multiple irradiations associated with ROS are scant. The RMFI of ROS in SAS-R cells was lower than that in parent SAS cells, and a slow decrease in RMFI levels was observed even after 1–3 d. HPF based detection of ROS indicated no significant changes in ROS in both SAS and SAS-R cells. Reportedly, CRR cells, such as SAS-R, rarely produce mitochondrial ROS [25]. DHR123 is a detection reagent for mitochondria-derived ROS [40]. It has also been reported that drugs such as 3-methyl pyruvate and dichloroacetate enhance radiosensitivity by increasing the production of mitochondria-derived ROS in cancer cells [41,42]. In this study, a small but statistically significant increase in RMFI by DHR123 was observed in SAS (*p* = 0.001) and SAS-R (*p* = 0.04) 2 d after irradiation. The increase in RMFI by DHE on day 3 after high-dose-rate PR of SAS may be due to the timing effect of superoxide exposure from mitochondria. The increase in RMFI induced by DHE on day 3 by high-dose-rate PR in SAS cells was thought to be due to superoxide from mitochondria, due to the timing of the increase. These results suggest that the cumulative effect of mitochondria-derived delayed ROS facilitates the enhancement of cytotoxic effects in high-dose-rate PR.

The results of this study indicated that high-dose-rate PR is an effective method that may be used to improve the sensitivity of radioresistant cells easily and quickly without involving drugs. It may expectedly improve the control rate of radiotherapy in clinical practice. In this study, we did not evaluate the effect on normal cells, as it is expected that the effects of high-dose-rate PR will be greater on tumor cells than in normal tissues in clinical practice. This is because our results suggest that the influence of the low repeated dose (LORD) effect is one of the mechanisms of high-dose-rate PR. In radiotherapy, radiation is delivered to tumors from multiple directions to reduce the dose to normal tissues, and thus tumors are irradiated more frequently compared to normal cells. We were able to clarify that the cytotoxic effect was enhanced using a high-dose-rate PR of standard 2 Gy fractionated radiation, or by increasing the dose up to 8 Gy, irradiating for 5 d, and performing a colony assay using a no-processing dish. In recent years, respiratory-gated radiotherapy, in which irradiation is performed at a certain respiratory phase of the respiratory cycle, and intensity modulated radiotherapy, which uses more field than conventional treatments, have resulted in multiple low dose irradiation procedures. Although there is some concern about the effect of extended treatment time associated with these high-precision radiotherapies, the results of this study suggest that the treatment effect is unlikely to be reduced unless the treatment time is significantly extended.

## 5. Conclusions

We showed that high-dose-rate PR with short time intervals may easily enhance a cytotoxic effect on radioresistant cells, using only the irradiation protocol. The *DMF*_10_ of SAS and SAS-R of high-dose-rate PR by the colony assay was about 1.1, indicating that the enhancement of cytotoxic effect by high-dose-rate PR was maintained even after five consecutive days of irradiation. The mechanism underlying high-dose-rate PR appears to involve DSB repair and increasing delayed ROS, as indicated by residual γH2AX remaining after 24 h.

## Figures and Tables

**Table 1:** Values of the parameters obtained from survival curves of SAS and SAS-R cells using the LQ model.

		α	β	α/β	*D*_10_	*DMF*_10_ ^b^
		(Gy^−1^) ^a^	(Gy^−2^) ^a^	(Gy) ^a^	(Gy)	
SAS	Single exposure	0.34 ± 0.03	0.034 ± 0.005	10.2 ± 1.9	4.6	1.00
	High-dose-rate PR	0.39 ± 0.012	0.038 ± 0.002	10.3 ± 0.7	4.2	1.11
SAS-R	Single exposure	0.00017 ± 0.06	0.061 ± 0.005	0.0028 ± 0.5	6.1	1.00
	High-dose-rate PR	0 ± 0.03	0.077 ± 0.004	0	5.5	1.12

^a^ Data represent the values ± standard error. ^b^ *DMF*_10_: dose-modification factor at 10% survival.
